# The role of rapid diagnostic tests in managing adults with pneumonia in low-resource settings

**DOI:** 10.15172/pneu.2014.5/444

**Published:** 2014-12-01

**Authors:** Stephen J. Aston

**Affiliations:** 13 Malawi Liverpool Wellcome Trust Clinical Research Facility, PO Box 30096, Chichiri, Blantyre 3, Malawi; 23 Liverpool School of Tropical Medicine, Liverpool, UK

**Keywords:** BinaxNOW *Streptococcus pneumoniae* antigen card, lipoarabinomannan, Xpert MTB/RIF, 1-3-β-D-glucan, pneumonia

## Abstract

In well-resourced settings the systematic use of rapid diagnostics tests (e.g. pneumococcal urinary antigen test) that define the causal pathogen to direct therapy has not resulted in significantly improved outcomes in adults withpneumonia. The management of pneumonia in many low-resource settings is complicated by a substantial burden of tuberculosis and HIV-associated opportunistic infections, in addition to the usual spectrum of pathogens seenin well-resourced settings. Clinical features alone do not reliably distinguish between these different aetiologiesand physicians often have to treat empirically. Given the limitations in diagnostic laboratory capability present inmost low-resource settings, rapid and point-of-care diagnostic tests could become valuable tools to guide treatment decisions. Pneumococcal and *Legionella* urinary antigen tests are specific and moderately sensitive, but their utilityin low-resource settings is uncertain. The Xpert MTB/RIF (Cepheid, USA) platform and rapid assays for urinary lipoarabinomannan can substantially speed up tuberculosis diagnosis; the current challenge is to translate this intoearlier treatment and hopefully improve patient outcome. In HIV-infected patients, 1-3-β-D-glucan is a serum marker of *Pneumocystis jirovecii* infection with excellent sensitivity. Further studies are needed to assess the clinical utilityand cost-effectiveness of these rapid diagnostic assays when they are incorporated into treatment algorithms.

## 1. Introduction

In well-resourced settings, rapid diagnostic tests to define the causative pathogen in community-acquired pneumonia (CAP) have failed to contribute significantly to patient management or improve outcomes [1]. In these settings, the spectrum of potential pathogens is generally well defined and empirical treatment algorithms are devised to reliably provide adequate antimicrobial cover [2]. Concordance to guidelines that define empirical treatment on the basis of severity, rather than tailoring of therapy on the basis of aetiological diagnostic tests, is important in achieving optimal outcomes [1]. The situation in low-resource settings is different. Frequently, the spectrum of pathogens is not well-defined; where data do exist, pathogens like *Mycobacterium tuberculosis* feature prominently [3]. In areas of high HIV-prevalence, such as most of sub-Saharan Africa, the majority of adults hospitalised with pneumonia are HIV-positive and are often highly immunocompromised, thus potentially harbouring a much broader range of pathogens [4]. Given this different landscape of disease, a pathogen-directed approach, at least to the level of distinguishing bacterial pneumonia from tuberculosis (TB) and *Pneumocystis jirovecii* or other opportunistic pathogens, is required. However, physicians in low-resource settings often only have access to the most rudimentary of laboratory diagnostic services. Point-of-care or rapid diagnostic tests to define the causative pathogen may potentially have great utility in these settings. This review discusses recent advances in point-of-care and rapid diagnostic tests for respiratory pathogens and their potential utility in low-resource settings, focusing particularly on areas of high HIV prevalence.

## 2. Pneumococcal pneumonia

*Streptococcus pneumoniae* is the most commonly identified causative organism of CAP in most settings [2, 3]. Classical microbiological techniques of establishing the diagnosis of pneumococcal pneumonia (e.g. sputum Gram-stain, sputum or blood culture) are highly specific, but insensitive and often slow [5]. The Alere BinaxNOW *Streptococcus pneumoniae* Antigen Card (BinaxNOW-SP; Alere, USA) is an immunochromatographic test for the presence of the pneumococcal C-polysaccharide coat protein in urine; the test can produce a result within 15 minutes of the specimen being obtained and requires minimal laboratory infrastructure or staff expertise to perform [6]. Accurate assessment of the diagnostic performance characteristics of BinaxNOW-SP has been hampered by the lack of a sensitive and specific reference standard test. A recent meta-analysis that used a latent class model approach to adjust for the variable and imperfect nature of the reference standard, estimated the sensitivity and specificity of BinaxNOW-SP for the diagnosis of *S. pneumoniae* infection in adults with CAP was 74% and 97%, respectively [5]. In practice, when used in conjunction with classical microbiological methods, the assay gives an incremental improvement in detection of pneumococcal pneumonia, particularly for patients already treated with antibiotics [6]. The mean duration of BinaxNOW-SP positivity is up to 8 weeks, hence it cannot be used as a test of cure and should not be used in cases of recurrent pneumonia less than 4 months after pneumonia caused by *S. pneumoniae* [6].

BinaxNOW-SP has poor specificity — and hence limited utility — in children owing to high rates of asymptomatic nasal carriage of *S. pneumoniae* [7]. In many low-resource settings there are also high rates of nasopharyngeal colonisation in adults [8, 9]. In a recent study of 98 healthy Thai women, *S. pneumoniae* nasopharyngeal colonisation (present in 25% of women) was not associated with positive BinaxNOW-SP [10]. In well-resourced settings, the sensitivity and specificity of BinaxNOW-SP are reportedly unaffected by HIV-status [11]. The combined effect of HIV-infection and pneumococcal nasopharyngeal colonisation — which is common in low-resource settings — on the specificity of the test has not yet been studied in detail.

Regardless of its performance characteristics, the practical value of a diagnostic test for pneumococcal pneumonia merits careful consideration (Table 1). Recommended empirical therapy always includes anti-pneumococcal coverage. A confirmed diagnosis may be desirable in order to justify narrowing the spectrum of therapy with the aim of reducing the risk of complications of broad spectrum antibiotics (e.g. *Clostridium difficile)* and decreasing the prevalence of community antibiotic resistance [5]. Conversely, the possibility of polymicrobial infection and the potential benefit of combination therapies in cases of severe pneumococcal pneumonia, support continued broad empirical treatment even when pneumococcal infection is confirmed [1, 12]. In a recent randomised trial, narrowing the spectrum of antibiotic treatment on the basis of pneumococcal and *Legionella* urinary antigen tests in adults hospitalised with CAP, failed to demonstrate any significant clinical or economic benefit and moreover was associated with a higher risk of clinical relapse [1]. In settings where the majority of patients hospitalised with pneumonia have HIV, and typically advanced immunosuppression, polymicrobial infection with not only other typical CAP pathogens but *M. tuberculosis* and other opportunistic pathogens is common [13, 14]. In this context, clinicians should have a low threshold for additional investigation, even after pneumococcal pneumonia is confirmed, if patients fail to improve or show clinical features highly suggestive of alternative aetiologies.

**Table 1:** Characteristics of the *Streptococcus pneumoniae* urinary antigen test (BinaxNOW-SP; Alere, USA) for the diagnosis of pneumococcal pneumonia

Advantages	Disadvantages
Urine specimen is easy to obtain and no processing needed Minimal laboratory infrastructure and operator training required Rapid results (≤15 minutes) High specificity Incremental improvement in sensitivity when combined with blood and sputum culture Remains positive after starting antibiotics Facilitates early targeting of antibiotic treatment	High cost Polymicrobial infection not identified No information on antimicrobial resistance Insufficient sensitivity to exclude pneumococcal pneumonia Early narrowing of antibiotic treatment not of proven benefit and may be deleterious

## 3. *Legionella* pneumonia

In well-resourced settings, Legionnaires’ disease is responsible for 1–5% of cases of CAP overall and a higher proportion of severe disease [15, 16]. Greater than 90% of reported cases of Legionnaires’ disease are caused by *Legionella pneumophilia* and amongst these over 80% are caused by serogroup 1 strains [15], although there is considerable geographical variation in the predominance of particular *Legionella* species and *L. pneumophilia* serotypes.

Microbiological diagnosis of *Legionella* pneumonia is difficult. Its fastidious growth requirements mean that sputum and particularly blood culture are insensitive. Until recently, serology was the commonest means of diagnosis with both acute and convalescent specimens normally required for confirmation [17, 18].

Approximately 80% of patients with *L. pneumophilia* serogroup 1 infection excrete bacterial antigen in urine [17, 18]. There are several available radio- and enzyme-immunoassays that detect urinary *Legionella* antigens with similar sensitivity and specificity, approximately 80% and 99%, respectively [17]. More recently, an immunochromatographic version of the assay, analogous to BinaxNOW-SP, has been developed (Alere Binax *Legionella* Urinary Antigen Card; Alere) with comparable performance characteristics [19]. All of these urinary antigen assays only reliably detect serogroup 1 strains; their utility is therefore dependent upon the relative local predominance of *L. pneumophilia* serogroup 1 infection.

The burden of *Legionella* amongst adults with pneumonia in most low-resource settings is uncertain. Available data from sub-Saharan Africa indicate a very low prevalence of disease [3]. Assuming a low prevalence, the widespread use of *Legionella* diagnostic tests is not an appropriate use of scant resource. Equally, empirical treatment for *Legionella* and other atypical pathogens for all patients with moderate and severe pneumonia, as is recommended in most well-resourced settings, has substantial resource implications. Rather than attempting individual patient diagnosis, sentinel surveillance programmes or intermittent aetiological studies using serological testing to estimate the local burden of disease are a more practical way of informing treatment guidelines in low-resource settings.

## 4. Tuberculosis

### 4.1 Burden and challenges

In low-resource settings, particularly in sub-Saharan Africa, TB is common amongst patients presenting with pneumonia [3]. In African post-mortem series, TB is the most prominent cause of death of HIV-positive adults with respiratory infection [13]. In these settings, TB diagnosis is still largely reliant on smear microscopy and chest radiography despite their limited diagnostic accuracy; in HIV-associated TB, approximately only a quarter of cases are smear-positive and up to a quarter have normal radiographs [20]. The consequent delayed diagnosis not only causes increased morbidity for the affected patients, but permits increased onward transmission to contacts. The ideal diagnostic test needs to be sufficiently simple, robust and rapid, that it may be performed at the point-of-care, and give immediate results that are of sufficient specificity to justify treatment, whilst having excellent sensitivity in both HIV-negative and -positive patients, including those with advanced immunosuppression [21]. The last decade has seen renewed momentum in the area of TB diagnostics with new assays being developed, assessed, and now being incorporated into clinical practice [22] (Table 2). The following section will describe the development and recommended use of the urinary lipoarabinomannan (LAM) and Xpert MTB/RIF (Cepheid, USA) assays.

**Table 2:** Diagnostic tests for pulmonary tuberculosis: current, under evaluation and in development

Test	Comments	Ref
Chest radiography	Used as adjunct to smear microscopy. No radiographic pattern diagnostic of TB and substantial inter-observer variation. Appearances vary with immune status in HIV-associated disease.	23
Sputum smear microscopy	Rapid and widely available; often sole diagnostic test. Sensitivity is poor, particularly in HIV-associated TB; increased by sputum concentration and fluorescence microscopy rather than ZN staining.	23
Sputum culture	Gold-standard diagnostic test; often restricted to reference centres in resource-limited settings. Automated liquid culture systems (e.g. BACTEC MGIT 960; Becton Dickinson, USA) are faster and more sensitive than solid culture techniques.	23
Xpert MTB/RIF (Cepheid, USA)	Fully automated NAAT platform; allows rapid detection of MDRTB. More sensitive than sputum smear microscopy, particularly in HIV-infection. High setup and running costs; needs uninterrupted electrical supply.	22
Line probe assay (e.g. GenoType MTBDR*plus* 2.0; Hain Lifescience GmbH, Germany)	NAAT mainly used for rapid identification of isoniazid mono-resistance and MDRTB in culture isolates. Now adapted for use on clinical specimens; comparable accuracy to Xpert MTB/RIF.	24
Loop-mediated isothermal amplification	Simplified, manual NAAT for use in basic laboratory settings. Currently in large scale evaluation; estimated sensitivity is 88% overall and 56% in sputum smear-negative TB.	25
Urinary lipoarabinomannan (e.g. Alere Determine TB LAM Ag; Alere, USA)	POC assay facilitating rapid treatment initiation. Most sensitive in advanced HIV-infection; incremental increase in sensitivity over sputum smear microscopy and Xpert MTB/RIF alone.	26, 27
Volatile organic compounds	Mass spectrographic analysis of exhaled breath for compounds associated with pulmonary TB. In early stages of development; requires sophisticated instrumentaion.	21
Serological tests	Humoral response to TB is highly variable; no single antibody measurement proved useful in diagnosis. Assays based on simultaneous detection of multiple antibodies in development.	21
Proteomics	Serum proteomic profile of active TB derived by mass spectrometry. Diagnostic tests based on candidate biomarkers in development.	21

TB, tuberculosis; ZN, Ziehl-Neelsen; NAAT, nucleic-acid amplification test; MDRTB, multi-drug resistant tuberculosis; POC, point-of-care.

### 4.2 Diagnostic assays

#### 4.2.1 Urinary lipoarabinomannan (LAM)

Quite apart from the insensitivity of smear microscopy, sputum specimens are frequently difficult to obtain; many patients hospitalised with pneumonia are unable to produce a specimen of adequate quality. Sputum induction or other invasive means of obtaining lower respiratory tract specimens are often unavailable in low-resource settings or contraindicated due to patient instability. Diagnostic assays based on urine are potentially advantageous for several reasons: collection is straightforward — in even the sickest of patients — and does not generate potentially hazardous aerosols; specimens are safe to handle and of consistent quality [28].

The mycobacterial cell wall glycopeptide, LAM, is one of several mycobacterial antigens detectable in urine during TB infection [26]. LAM is released in large quantities from metabolically active or degrading cells and enters the systemic circulation [29] from disease sites or during mycobacteraemia and is subsequently filtered at the glomerulus to enter the urine [26, 30, 31].

The first proof-of-principle assay for the detection of urinary LAM was developed by Hamasur *et al* [32]; urine specimens required extensive processing before being used in a 96 well-plate direct capture enzyme-linked immunosorbent assay (ELISA) format to detect LAM. Initial clinical evaluations demonstrated a sensitivity of 74% and specificity of 87% [33]. A similar assay developed by Chemogen Inc. (USA) for use with unprocessed urine showed similar sensitivity and improved specificity in a study of Tanzanian patients with suspected TB [34]. Further commercial versions of the assay have been developed (MTB ELISA; Chemogen Inc. and Clearview TB ELISA; Alere) and extensively evaluated in studies in Africa and Asia and a variety of clinical settings. The early promising results, however, have not been replicated; in meta-analyses, the pooled estimates of sensitivity of MTB ELISA and Clearview TB ELISA is 45% and 40%, respectively [26, 27]. Reported specificities have also varied considerably, ranging from 88% to 100%, possibly reflecting differences in the reference standard diagnostic test used or the occurrence of disseminated disease [26]. All recent studies indicate a clear difference in the sensitivity of urinary LAM assays according to HIV-status; incremental improvement in sensitivity ranges from 3% to 53% in HIV-positive subgroups [27]. The sensitivity also increases with the degree of immunosuppression such that it is as high as 85% in patients with CD4 counts less than 50 cells/µL; this is considerably greater than that of sputum smear microscopy in this patient group [26, 27].

The latest iteration of the LAM assay (Alere Determine TB LAM Ag; Alere) is a simplified lateral flow version [26]. This genuinely point-of-care test uses a small volume of unprocessed urine, gives results in around 30 minutes and has comparable performance characteristics to Clearview TB ELISA [35]. The test kits are relatively cheap, may be stored at room temperature and require minimal training to use [26]. Two large scale evaluations of the Alere Determine TB LAM Ag assay have helped to further define its role in TB diagnosis [35, 36]. Not only is sensitivity greatest in patients with a low CD4 count (67% if CD4 <50 cells/µL), it is also significantly higher when other features of severe disease are present (i.e. advanced symptoms, haemoglobin <8 g/dL, C-reactive protein [CRP] >200 mg/L] [20]. There is an incremental increase in sensitivity when urinary LAM assays and sputum smear microscopy are used in combination, indicating that these tests may detect different groups of TB patients [27, 35].

Urinary LAM assays are an important development in diagnostics for HIV-associated TB. Patients hospitalised with pneumonia in particular are a suitable target group for their use since they are likely to have the features of advanced immunosuppression and severe disease which are associated with the highest degree of assay sensitivity. In this group, a urinary LAM assay may function as useful rule-in test to facilitate rapid treatment initiation amongst some TB patients with the highest risk of mortality [37].

#### 4.2.2 Xpert MTB/RIF

The development of the Xpert MTB/RIF platform is the single-most important recent development in TB diagnostics and has been extensively reviewed elsewhere [22]. This fully integrated and automated diagnostics platform is capable of identifying *M. tuberculosis* complex and the presence of mutations conferring rifampicin resistance in unprocessed sputum specimens in less than 2 hours [30]. World Health Organization (WHO) endorsement in 2010 and subsequent innovative funding strategies have permitted the widespread rollout of the Xpert MTB/RIF platform to many low- and middle-income countries and ensured its position as a central pillar of TB diagnostics in these settings [22].

The Xpert MTB/RIF has been extensively evaluated to determine its real-life diagnostic performance characteristics, operational feasibility and cost-effectiveness. In a recent Cochrane systematic review including 18 studies that compared Xpert MTB/RIF against sputum culture, the overall pooled sensitivity was 88% and specificity was 98% [38]. The estimated sensitivity for sputum smear-positive and smear-negative subgroups was 98% and 67%, respectively [38]. In HIV-associated TB, the sensitivity of Xpert MTB/RIF is estimated at 84% and is consistently better than sputum smear microscopy with a median increment of 30% [22].

The implementation of TB diagnostic algorithms based on Xpert MTB/RIF in low-resource settings has presented considerable challenges. Cost, training needs and other logistical constraints have to date largely restricted the rollout of the Xpert MTB/RIF platform to central laboratories. In settings of poor infrastructure, this separation of the patient and test result may impede the timely initiation of treatment [22]. A recent study demonstrated, however, that the Xpert MTB/RIF system can be accurately administered by a trained nurse in primary health-care centres to facilitate improved rates of same-day diagnosis and treatment initiation [39]. Future efforts will be focused on further rollout and decentralisation of the Xpert MTB/RIF system.

## 5. *Pneumocystis jirovecii* pneumonia

*P. jirovecii* pneumonia (PCP) is one of the commonest HIV-related opportunistic infections in well-resourced settings and should be considered in all HIV-positive patients presenting with features of respiratory infection [40]. Historically, definitive diagnosis has relied on direct visualisation of *Pneumocystis* organisms in bronchoalveolar lavage fluid and transbronchial tissue biopsy specimens (Table 3). Bronchoscopy is not routinely available for clinical care in most low-resource settings and so the burden of disease is unclear and traditionally assumed to be low [40–42]. However, several research bronchoscopy studies from African centres have demonstrated a considerable burden of PCP amongst HIV-infected patients with features of respiratory infection [43–46]. An effective point-of-care test for PCP remains some way off, but there has been some relevant progress that merits discussion. In recent years, molecular assays have begun to supersede direct visualisation techniques for the detection of *P. jirovecii* [50]. The improved sensitivity of molecular assays based on polymerase chain reaction (PCR) may permit the use of non-invasive specimens (where *Pneumocystis* organisms are present in lower concentrations) such as expectorated sputum [47], nasopharyngeal aspirates [52] and oral washes [49] for the diagnosis of PCP, thus removing the need for bronchoscopy. The laboratory infrastructure required for these molecular assays in their current format still, however, precludes their use in most low-resource settings. Recently recognised serum markers indicative of *Pneumocystis* infection are an alternative diagnostic strategy for PCP that may be more feasible in the short-term [53, 54].

**Table 3:** Summary of diagnostic tests for *Pneumocystis jirovecii* pneumonia (PCP)

Test	Comments	Ref
Microscopy		45, 46
Cell wall stains (e.g. Toluidine Blue O, calcofluor white)	Identification of cysts or trophozoites in BALF or lung biopsy specimens; ‘gold-standard’ diagnostic method.	
Trophozoite stains (e.g. Grocott’s methenamine silver, Diff-Quick)	Immunofluorescence more sensitive than cytochemical stains. Only suitable for use with bronchoscopic or induced-sputum specimens.	
Immunofluorescence	Training needed for accurate and consistent reporting.	
Molecular assays		47–50
PCR	Several genetic loci evaluated (e.g. mtLSUrRNA, HSP70, ITS, DHFR, MSG).	
nPCR	High assay sensitivity may lead to false positive result if colonised with Pneumocystis; new quantitative assays have improved specificity.	
qPCR	May be used with oral wash and upper respiratory tract specimens with reasonable sensitivity.	
Serum assays		
1-3-β-D-glucan	Cell wall component of Pneumocystis; highly sensitive marker of PCP. Other invasive fungal infections and some antibiotics may cause false positive results.	51, 52
S-adenosylmethionine	Essential metabolic intermediate; scavenged from host during active Pneumocystis infection such that serum levels are depleted. Poor diagnostic capability.	53

BALF, bronchoalveolar lavage fluid; PCR, single-round polymerase chain reaction; nPCR, nested PCR; qPCR, quantitative (real-time) PCR; mtLSUrRNA, mitochondrial large subunit rRNA; HSP70, heat shock protein 70; ITS, internal transcribed spacers; DHFR, dehydrofolate reductase; MSG, multicopy major surface glycoprotein; PCP, *Pneumocystis jirovecii* pneumonia

1-3-β-D-glucan is a common component of the cell wall of most pathogenic fungi, including *Pneumocysis*, and is released into the systemic circulation and detectable in serum during infection [56]. Several retrospective studies and a single prospective study are consistent in indicating that serum 1-3-β-D-glucan has excellent (>95%) sensitivity and reasonable specificity (84–86%) for detecting PCP in patients with both HIV and other forms of immunocompromise [54, 57]. This degree of sensitivity is sufficient to screen patients with a low or moderate pre-test probability of disease, such that a negative result could reasonably be used to exclude PCP [54]. There are many alternative causes of an elevated serum 1-3-β-D-glucan that are clearly relevant during acute illness in an immunocompromised patient, including other invasive fungal infections (e.g. histoplasmosis, candidiasis), use of β-lactam antimicrobials obtained from *Pencillium* species, Gram-negative endotoxinaemia and renal failure [58]. PCP also frequently co-exists with other pathogens such that a positive diagnosis may not justify narrowing the spectrum of treatment or discontinuing investigation [45]. Nonetheless, a diagnostic test for PCP with the precision of 1-3-β-D-glucan would represent a marked improvement on the empiricism that is currently practised in most low-resource settings where other diagnostic modalities are generally unavailable. The use of 1-3-β-D-glucan or other serum markers of PCP infection should be further assessed, and if shown to be promising, attempts made to develop assay formats that are adapted for use in low-resource settings [53].

## 6. Considerations for use of rapid diagnostic assays

The potential utility of point-of-care and rapid diagnostic tests for pneumonia in low-resource settings will relate to how frequently their results indicate a change in patient management and whether this change results in improved outcome. Recommendations for the use of diagnostic tests will vary between settings in line with the local prevalence of particular pathogens, the burden of HIV, and also the availability of alternative diagnostic tests. In the context of limited resources, diagnostic test use must be rationally targeted to specific patient groups such as those individuals with high severity disease or those that fail to improve with first-line treatment regimens.

**Figure 1:**
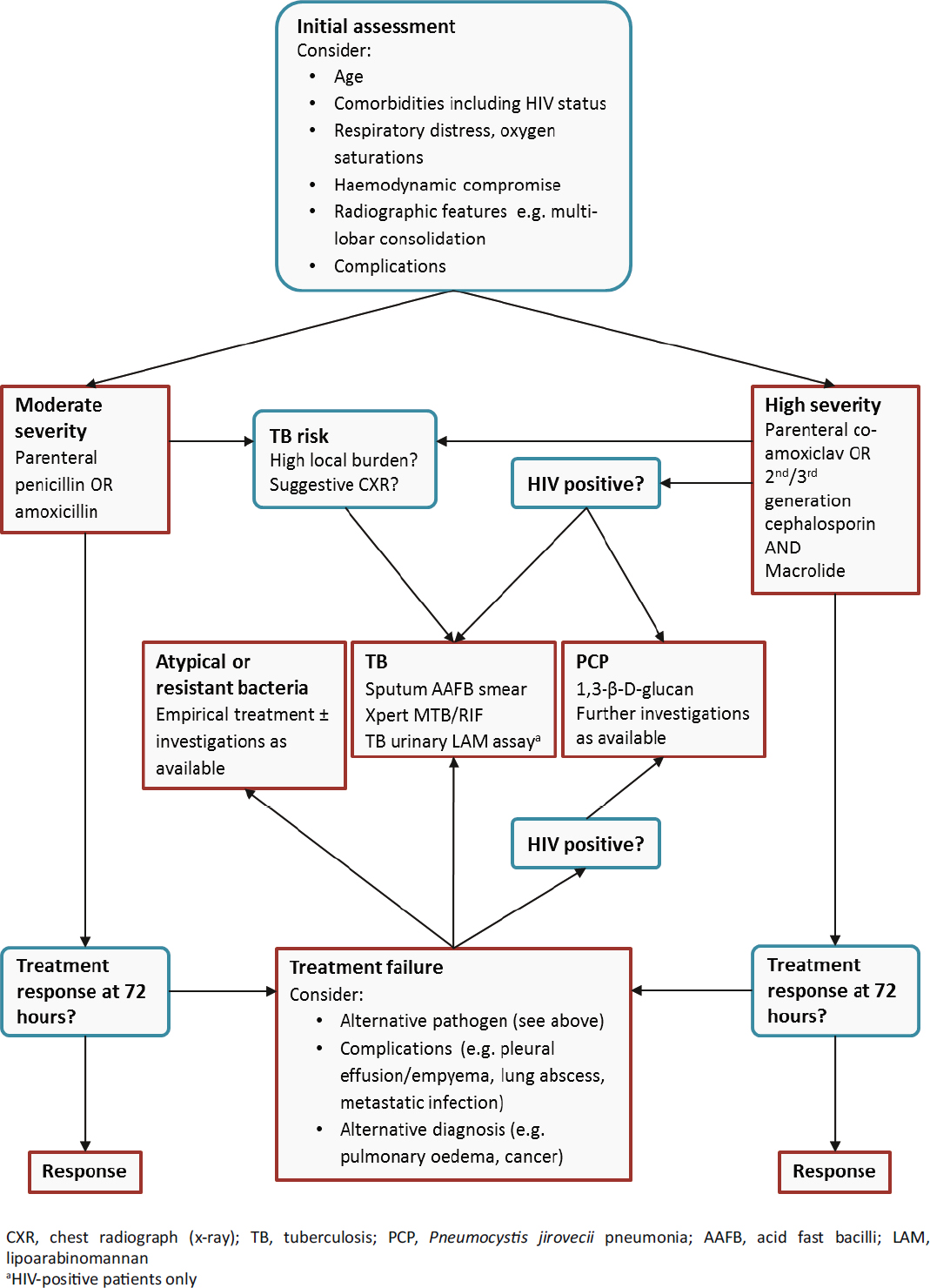
An algorithm for the investigation of adults hospitalised with pneumonia in low-resource settings. Initial antimicrobial choice is determined following an assessment of disease severity. Initial investigations are selected on the basis of disease severity, HIV status, and the clinical likelihood of TB. Further investigations are performed in patients that fail to respond to initial treatment. This algorithm should be considered in conjunction with clinical features; empirical treatment for TB or PCP may be appropriate.

A pneumonia management algorithm relevant to a low-resource setting that incorporates the use of rapid diagnostic tests is proposed in Figure 1. In this context, confirmation of pneumococcal aetiology by a urinary antigen test is unlikely to be a priority since the result will not substantially alter antimicrobial choice and it may not be an appropriate basis on which to curtail further diagnostic testing for TB. Urinary *Legionella* antigen tests are likely to be unaffordable in most low-resource settings; if used at all, they should be restricted to settings with a known burden of Legionellosis and for patients that fail to improve with first-line treatment. Assuming further positive evaluations, 1,3-β-D-glucan may have a role in diagnosing PCP in the absence of other diagnostic tests.

## 7. Conclusion

In many low-resource settings the management of adults with pneumonia is complicated by a high incidence of TB and other HIV-associated opportunistic infections. This broad range of pathogens is encountered in settings where diagnostic microbiology laboratory capabilities are weak. The use of point-of-care and other rapid diagnostic tests that require minimal laboratory infrastructure to perform may provide a feasible adjunct to guide the management of pneumonia in low-resource settings. Further evaluation is needed to determine not only the diagnostic precision of rapid diagnostic tests in low-resource settings, but also whether management algorithms that incorporate and alter management on the basis of their results are associated with improved patient outcome.
